# Polymorphisms near *EXOC4 *and *LRGUK *on chromosome 7q32 are associated with Type 2 Diabetes and fasting glucose; The NHLBI Family Heart Study

**DOI:** 10.1186/1471-2350-9-46

**Published:** 2008-05-22

**Authors:** Jason M Laramie, Jemma B Wilk, Sally L Williamson, Michael W Nagle, Jeanne C Latourelle, Jennifer E Tobin, Michael A Province, Ingrid B Borecki, Richard H Myers

**Affiliations:** 1 Department of Neurology, Boston University School of Medicine, Boston, MA, USA; 2 Bioinformatics Program, Boston University, Boston, MA, USA; 3 The Section of Preventive Medicine and Epidemiology, Department of Medicine, Boston University School of Medicine, Boston, MA, USA; 4 Department of Anatomy and Neurobiology, Boston University School of Medicine, Boston, MA, USA; 5 Center for Human Genome Sciences, Washington University School of Medicine, St. Louis, MO, USA

## Abstract

**Background:**

The chromosome 7q32 region is linked to metabolic syndrome and obesity related traits in the Family Heart Study. As part of a fine mapping study of the region, we evaluated the relationship of polymorphisms to fasting glucose levels and Type 2 diabetes.

**Methods:**

Thirty-nine HapMap defined tag SNPs in a 1.08 Mb region and a novel deletion polymorphism were genotyped in 2,603 participants of the NHLBI Family Heart Study (FHS). Regression modeling, adjusting for BMI, age, sex, smoking and the TCF7L2 polymorphism, was used to evaluate the association of these polymorphisms with T2D and fasting glucoses levels.

**Results:**

The deletion polymorphism confers a protective effect for T2D, with homozygous deletion carriers having a 53% reduced risk compared to non-deleted carriers. Among non-diabetics, the deletion was significantly associated with lower fasting glucose levels in men (p = 0.038) but not women (p = 0.118). In addition, seven SNPs near the deletion were significantly associated (p < 0.01) to diabetes.

**Conclusion:**

Chromosome 7q32 contains both SNPs and a deletion that were associated to T2D. Although the deletion region contains several islands of strongly conserved sequence, it is not known to contain a transcribed gene. The closest nearby gene, *EXOC4*, is involved in insulin-stimulated glucose transport and may be a candidate for this association. Further work is needed to determine if the deletion represents a functional variant or may be in linkage disequilibrium with a functional mutation influencing *EXOC4 *or another nearby gene.

## Background

Type 2 diabetes (T2D) is characterized by hyperglycemia due to insulin resistance and is accompanied by a failure of β cells to produce sufficient insulin. In the United States, the prevalence of diabetes has risen 40% from 1990 to 1999 [1]. This increase in T2D has been attributed to the recent rise in obesity. The correlation between diabetes and obesity has been shown in numerous epidemiological studies [2,3] although the mechanisms underlying this phenomenon are largely unknown. One current hypothesis proposes that malnutrition in the fetus can lead to the developmental impairment of pancreatic β cells, termed a "thrifty phenotype" and that later in life, these children are more susceptible to diabetes [4].

Candidate gene studies in T2D have implicated numerous gene variants that decrease disease risk, such as *PPARG *(P12A) [5], and others that increase disease risk, such as *KCNJ11*(E23K) [6]. One of the strongest T2D-associated loci has been mapped to the transcription factor *TCF7L2 *[7], (odds ratio ≈1.7) and this association has been replicated in numerous subsequent studies [8-20].

In addition to single nucleotide polymorphisms (SNPs), genomic insertion/deletion polymorphisms may also influence disease risk. One such example is the deletion polymorphism in the angiotension-converting enzyme (ACE), which has been shown to confer increased risk of coronary artery disease [21]. Recently the HapMap genotype data was used to search for segregating deletions by examining physically clustered failed SNP genotype assays, Mendelian inconsistencies, and departures from Hardy-Weinberg disequilibrium [22]. Five hundred and forty-one deletions were identified ranging in size from 1 to 754 kb. One predicted 10.3 kb deletion polymorphism was located under a widely replicated obesity linkage peak on chromosome 7q22-q36 [23-32] between the *EXOC4 *and *LRGUK *genes. Importantly, linkage to metabolic syndrome has also been reported for the Family Heart Study cohort in the 7q32 region [33].

The aim of this study was to examine SNPs and a chromosomal deletion on chromosome 7q32 in a sample of families exhibiting linkage to obesity and metabolic syndrome in the region [27,33]. Due to the presence of a gene (*EXOC4*) that is part of the exocyst complex (Exo70), which is involved in insulin-stimulated glucose transport, association to T2D risk and blood glucose levels was hypothesized. In addition, we examined the association between the minor allele of the SNP rs7903146 [7] within the transcription factor *TCF7L2 *and T2D disease risk. Each polymorphism's association to T2D risk was examined in a large Caucasian subset of the Family Heart Study (FHS) population comprising 2,396 participants (205 T2D cases). In addition to T2D disease affection status, fasting plasma glucose (FPG) was also examined among non-diabetics.

## Methods

### Subjects

The National Heart, Lung and Blood Institute (NHLBI) Family Heart Study (FHS) recruited families from four existing study centers located in Forsyth County, NC; Framingham, MA; Minneapolis, MN; and Salt Lake City, UT. Approximately one half of the families recruited from these study centers were at high risk for coronary heart disease (CHD) while the other half were selected randomly from their respective study populations.

The body mass index (BMI) SNP fine mapping study genotyped 2,421 FHS participants including 158 diabetics. For the deletion study, 416 of these, including two diabetics, either did not genotype for the deletion or did not have sufficient DNA for the deletion study. Therefore, an additional 182 FHS samples, including 49 diabetics, not originally included in the SNP analysis were typed for the deletion. In total, 2,603 participants, including 207 diabetics, were studied.

T2D diabetes was defined by self-report of diabetes diagnosis and limited to those with an age at diagnosis greater than 25. The controls used were participants recruited as part of FHS who did not report a diagnosis of diabetes. An enzymatic (glucose-oxidase) method (Kodak Ektachem 700 Analyzer, Rochester, NY) was used to measure fasting serum glucose as mg/dL. This study was approved by the institutional review boards (IRB) of the participating institutions and appropriate informed consent was obtained.

### Deletion Detection

Deletions were detected in the study population using real-time polymerase chain reaction (RT-PCR). To accurately type deletion variants, we designed primers to amplify regions within the proposed chromosomal deletion on chromosome 7q32. We performed TaqMan RT-PCR assays, using a VIC-labeled probe for a known diploid gene *PMP22 *(NM_153321) as a control reference and a VIC-labeled probe (Applied Biosystems, Foster City CA) for the experimental region, each run simultaneously. Each DNA sample was run in quadruplicate for each TaqMan assay on the PRISM^® ^7900 HT Sequence Detection System. The cycles-to-threshold (C_t_) was determined for each assay separately, and the difference between the average Ct for the experimental probe and control assays (ΔC_t_) was used to infer the presence of zero, one or two copies of the deleted segment. For the examined FHS study sample, the average ΔC_t _values clustered into three discrete groups, including one group showing amplification of the control locus and no amplification of the experimental locus. Treating each genotype cluster as '+/+' (wildtype), '+/-' and '-/-'DNA samples could be assigned a standard genotype (i.e. 11, 12, 22). A small number of individuals whose ΔC_t _value fell outside of the three genotype clusters (*n=*69, 2.8%) were coded as missing genotypes.

### SNP Genotyping

SNPs in the genes neighboring the deletion were genotyped as part of a fine-mapping study of BMI. Thirty-nine Tag SNPs were selected using the HapMap tagger algorithm in the region between 132,552,341 (rs6467475) and 133,619,534 (rs1421483). In addition, the SNP rs7903146, located on chromosome 10 at 114,748,089 bp within an intron of the gene *TCF7L2 *(NM_030756), was genotyped. The TCF7L2 SNP was typed using the TaqMan^® ^technology developed by Applied Biosystems (Foster City, CA) using the PRISM^® ^7900 HT Sequence Detection System. The 39 HapMap derived SNPs were genotyped using the Illumina Golden Gate^® ^assay method, through the Illumina Fast-Track Genotyping service. Mendelian inconsistencies were identified using INFER within the PEDSYS software package [34], and genotypes in the pedigrees where inconsistencies were found were removed.

### Linkage Disequilibrium (LD) Assessment

We assessed the LD between the chromosomal deletion obtained by RT-PCR, using deletion genotypes ('+/+', '+/-', '-/-') coded as 11, 12 and 22 and adjacent SNPs. The software program Haploview [35] was used to estimate the pairwise LD (*r*^2^) between the chromosomal deletion and nearby SNPs within 500 kb.

### Statistical Analysis

To evaluate the relationship of polymorphisms to T2D, dominant and recessive modeling of the minor allele was performed in a logistic regression implemented with a generalized estimating equation and adjusted for TCF7L2 minor allele, BMI at age 25 (based on participants' self report), study center, age, age^2^, age^3^, sex, and smoking history (never/ever). The relationship to T2D for the *TCF7L2 *SNP (rs7903146) was modeled as a dominant genetic effect (major homozygotes = 0, heterozygotes and minor homozygotes = 1) as previously reported [7] and the deletion polymorphism modeled as a recessive genetic effect ('+/+ wildtype' and '+/-' = 0 and '-/-' = 1) using the same covariates described above. As the FHS sample included prevalent diabetics, analyses of diabetes status were adjusted for an age variable that was defined as the age at diagnosis for diabetic cases and the age at clinical examination for non-diabetic controls. No correction for multiple testing was used in these analyses and, therefore, all p-values are reported as nominal p-values.

In addition to diabetes status, fasting glucose (mg/dL) levels were analyzed in non-diabetics using a linear GEE regression model adjusted for BMI, study center, age, age^2^, age^3^, smoking history status and sex. The deletion and TCF7L2 polymorphism were analyzed together in the same model. In this regression model, measurements of BMI and age at the time of examination were used.

## Results

Clinical characteristics of the study subjects are shown in Table 1. The mean age at examination of the diabetics was 61.57 with a range from 25.6 to 84.7 and of the non-diabetics participant's mean age was 52.0 with a range from 25.2 to 91.0. Both the BMI at examination (*p *= 10^-4^) and the reported BMI at age 25 (*p *< 10^-4^) were significantly different between diabetics and non-diabetics. However, the age at examination of non-diabetics was not different from the age at diagnosis of diabetes among the diabetics (*p *= 0.82).

**Table 1:** Characteristics of the study population

	Diabetics	Non-diabetics
	
*N *genotyped for deletion	205	1982
*N *genotyped for SNPs	158	2263
Total studied for either SNPs or deletion*	207	2396
Male	55.1%	46.7%
Age at onset of diabetes (years)	51.7 ± 11.6	-
Age at examination (years)	62.2 ± 9.9	51.8 ± 13.4**
BMI (kg/m^2^) at examination	30.0 ± 5.1	27.5 ± 5.8**
BMI (kg/m^2^) at age 25	24.5 ± 4.9	22.9 ± 3.6**
Fasting glucose (mg/dL)	173.9 ± 70.1	95.4 ± 16.1**
Center (%):		
Forsyth County, North Carolina,	22.2	22.5
Minneapolis, Minnesota	24.7	28.0
Framingham, Massachusetts	19.8	22.0
Salt Lake City, Utah	33.3	27.5

*means and frequencies correspond to sample with either deletion or SNP genotyping

** p < 10^-4^

### Deletion Polymorphism

**Figure 1:**
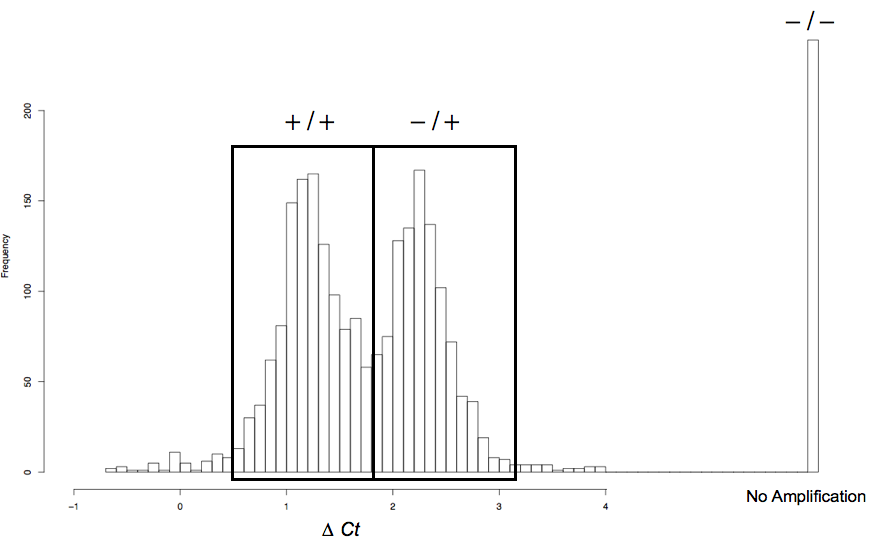
**Insertion/deletion genotype clusters**. Deletion genotype clusters as measured by real-time PCR. Individuals outside of the clusters, defined by the black boxes, were marked with an unknown genotype.

Table 2 lists the sequences for the control (*PMP22*) and deletion detection primers and probes. A total of 2,198 study participants were assayed and the average ΔC_t _was used to generate three genotype clusters (Figure 1). The deletion was present in 52.7% (*n=*1,152) of the study population with a deletion allele frequency of 31.2% and was in Hardy-Weinberg equilibrium (*p *= 0.85).

**Table 2:** Primer and probe sequences used in the RT-PCR deletion assay

Gene	Region		
PMP22 (Control)	chr17:15,074,941–15,075,005		
		Primer 1	CCCTTCTCAGCGGTGTCATC
		Primer 2	ACAGACCGTCTGGGCGC
		Probe	VIC – TTCGCGTTTCCGCAAGAT
	chr7:133,441,108–133,441,133		
		Primer 1	GCCTTGCCCGAGTACATATT
		Primer 2	AGAGTTGGCCTCTGTCCCTA
		Probe	VIC-CAGCTGGTGTTACCAGTAAAGGCCCT

Diabetes risk results for the deletion versus the TCF7L2 polymorphism are shown in Table 3. In the total study sample, there was a protective effect of the homozygous deletion genotype on diabetes (*p *= 0.016 odds ratio (OR) = 0.47, 95% CI 0.25–0.87). In addition, BMI at age 25 was shown to be a strong risk factor for diabetes (*p *< 10^-4^, beta estimate = .09, OR for a 1 unit BMI increase at age 25 = 1.09). In the total study sample, 5.4% (*n *= 11) of the diabetics were homozygous for the deletion as compared to 10.2% (*n *= 203) of the non-diabetics.

**Table 3:** Logistic GEE results incorporating both polymorphisms

Diabetes Status				
	Homozygous deletion		rs7903146 minor allele	
		
	OR [95% CI]	*p-value*	OR [95% CI]	*p*-value
				
Both sexes	0.47 [0.25,0.87]	0.016	1.31 [0.95,1.81]	0.099
Women	0.57 [0.22,1.52]	0.26	1.63 [1.02,2.59]	0.039
Men	0.45 [0.20,1.05]	0.064	1.14 [0.76, 1.72]	0.53

Results from logistic GEE models incorporating the deletion genotyping and rs7903146, including sex-stratified results. Odds ratios (OR), 95% confidence interval (95% CI) and *p*-values are reported.

*205 cases, 1982 controls – adjusted for BMI at age25, onset age for cases (w/square and cubed)

When stratified by sex, the protective effect of the deletion on diabetes was stronger in men (*p *= 0.064, OR = 0.45, 95% CI 0.2–1.05) than in women (*p *= 0.26, OR = 0.57, 95% CI 0.22–1.52) with BMI at age 25 remaining a strong risk factor for diabetes in each gender (*p *< 10^-4^). In men, 5.4% (*n *= 6) of the diabetics were homozygous for the deletion as compared to 11.4% (*n *= 107) of the non-diabetics. In women, the individuals homozygous for the deletion represented 5.4% (*n *= 5) of the diabetics and 9.2% (*n *= 96) of the non-diabetics, although the effect was not statistically significant.

In addition, we tested the effect of the deletion and the *TCF7L2 *SNP on fasting glucose levels among non-diabetics stratified by sex (see Table 4). In an analysis of both sexes combined, the TCF7L2 minor allele was associated with a 1.3 mg/dL higher mean glucose level (p = 0.045), whereas the deletion polymorphism did not have a significant effect on glucose levels. Men homozygous for the deletion polymorphism had a statistically significant decrease in fasting glucose levels (*p *= 0.038, β-estimate = -2.57 mg/dL) while the *TCF7L2 *SNP had a modest increase in fasting glucose levels (*p *= 0.089, β-estimate = 1.91 mg/dL). Neither polymorphism had a significant effect on fasting glucose in women.

**Table 4:** GEE results for fasting glucose levels (mg/dl)

	Homozygous deletion		rs7903146 minor allele	
		
	beta-estimate	*p-value*	Beta-estimate	*p*-value
				
Both sexes	-0.50	0.57	1.33	0.045
Women	1.89	0.118	0.77	0.283
Men	-2.57	0.038	1.91	0.089

Results from GEE models for the deletion genotyping and rs7903146 modeled together including sex-stratified results.

### SNP Association

Association analyses to diabetes using SNPs in the *EXOC4 *and *LRGUK *gene regions identified multiple polymorphisms with evidence for association (Table 5). Seven SNPs (rs3823572, rs12531707, rs11770757, rs7457999, rs6953590, rs12670589, and rs1421483) demonstrated significant association (p = 0.01) to fasting glucose. Using a dominant modeling of the minor allele, the SNP rs12531707 in an *EXOC4 *intron produced an odds ratio for diabetes of 1.79 (p = 0.009). The SNP in strongest LD with the deletion, rs7457999, exhibited a protective effect for diabetes. Using a recessive modeling of the minor allele, the SNP rs12670589 in a *LRGUK *intron produced an OR for diabetes of 2.02 (p = 0.002). Other SNPs in both *EXOC4 *and *LRGUK *produced larger ORs for the recessive model, but the results were based on a small number of homozygous minor allele carriers. For example, rs11770757 produced an OR = 11.9 based on two homozygous minor allele carriers each in cases and controls, and the results may be spurious. We have presented all recessive results for which the model converged in the hopes that replication studies in larger samples of diabetics will examine these SNPs.

**Table 5:** SNP association to diabetes in region surrounding deletion

SNP	bp position	Gene	Dominant OR	Dominant p-value	Recessive OR	Recessive p-value
rs6467475	132552341		1.04	0.85	1.29	0.30
rs11979455	132558191		1.22	0.24	1.46	0.20
rs6979285	132564427		0.70	0.22		
rs6951889	132566505		0.73	0.09	0.90	0.77
rs10262862	132606574	*EXOC4*	1.03	0.88	1.36	0.19
rs13242614	132616839	*EXOC4*	0.93	0.76	2.67	0.03
rs1922420	132647772	*EXOC4*	0.94	0.74	1.07	0.86
rs6978272	132738579	*EXOC4*	1.03	0.88	0.76	0.71
rs13241123	132786047	*EXOC4*	0.83	0.29	0.79	0.42
rs6971417	132816709	*EXOC4*	1.45	0.04	1.42	0.33
rs13237737	132842959	*EXOC4*	1.15	0.71	4.00	0.18
rs10755879	132910055	*EXOC4*	0.71	0.14	2.10	0.17
rs6954842	132938673	*EXOC4|KIAA1699*	1.19	0.35	1.23	0.42
rs1362736	133060409	*EXOC4|KIAA1699*	1.00	1.00	1.59	0.47
rs17167240	133075208	*EXOC4|KIAA1699*	0.83	0.34	0.44	0.11
rs9649047	133084054	*EXOC4|KIAA1699*	0.98	0.93	1.26	0.72
rs11772444	133104414	*EXOC4|KIAA1699*	1.51	0.02	1.72	0.11
rs13222377	133116886	*EXOC4|KIAA1699*	0.99	0.97	0.89	0.79
rs17167267	133119649	*EXOC4|KIAA1699*	1.15	0.56		
rs748754	133159609	*EXOC4|KIAA1699*	1.09	0.60	0.81	0.52
rs12155007	133235969	*EXOC4|KIAA1699*	0.74	0.31	4.79	0.05
rs4266574	133277976	*EXOC4|KIAA1699*	0.80	0.24	0.29	0.04
rs2971970	133294318	*EXOC4|KIAA1699*	1.42	0.07	1.48	0.25
**rs3823572**	133331141	*EXOC4|KIAA1699*	0.89	0.53	0.58	**0.01**
**rs12531707**	133378771	*EXOC4|KIAA1699*	1.79	**0.009**	1.15	0.49
rs6955114	133387570	*EXOC4|KIAA1699*	1.26	0.20	0.97	0.90
rs6971064	133390804	*EXOC4|KIAA1699*	1.17	0.40	0.87	0.81
**rs11770757**	133398486	*EXOC4|KIAA1699*	0.71	0.33	11.87	**0.006**
**rs7457999**	133398775	*EXOC4|KIAA1699*	0.62	**0.01**	0.58	0.22
rs10246346	133415861		1.04	0.86	0.79	0.76
Deletion						
rs13246630	133472159	*LRGUK*	1.36	0.09	1.27	0.27
**rs6953590**	133487840	*LRGUK*	1.52	**0.01**	1.86	**0.006**
rs17761994	133494082	*LRGUK*	1.23	0.35		
**rs12670589**	133495637	*LRGUK*	1.30	0.14	2.03	**0.002**
rs892984	133502721	*LRGUK*	0.64	0.10	0.92	0.92
rs1222430	133566300	*LRGUK*	0.73	0.24	1.08	0.93
rs1421477	133569572	*LRGUK*	1.15	0.51		
rs1450890	133584737	*LRGUK*	0.86	0.41	1.31	0.33
**rs1421483**	133619284		1.01	0.97	3.93	**0.009**

Within the total study population the SNP rs7903146, located within an intron of the gene *TCF7L2 *(NM_030756), had a minor allele frequency of 31%. In a dominant model, the *TCF7L2 *SNP was modestly associated with diabetes risk (*p *= 0.099, OR = 1.31, 95% CI 0.95–1.81). In the total study sample, 57.5% (*n *= 119) of the diabetics were minor risk allele carriers as compared to 51.9% (*n *= 1243) of the non-diabetics.

When stratified by sex, the minor allele of the *TCF7L2 *SNP was associated with a large increased risk for T2D in women (*p *= 0.039, OR = 1.63, 95% CI 1.02–2.59) whereas no effect was seen in men (*p *= 0.53, OR = 1.14, 95% CI 0.76–1.72). In women, 63.4% (*n *= 59) of the diabetics were minor allele risk carriers compared to 50.6% (*n *= 646) of the non-diabetics. In men, 52.6% (*n *= 60) of the diabetics were minor allele risk carriers compared to 52.6% (*n *= 597) of the non-diabetics.

**Figure 2:**
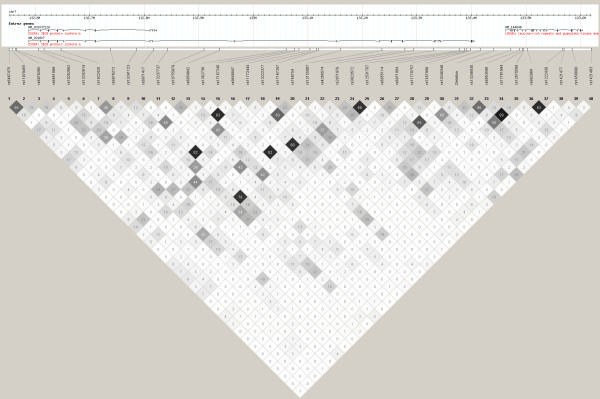
**LD structure surrounding the insertion/deletion polymorphism**. Linkage disequilibrium (*r*^2^) of the deletion polymorphism ('Deletion') and surrounding genotyped SNPs within the FHS study population. The legend above the LD plot shows the location of the genes *EXOC4 *and *LRGUK *(FLJ32786).

Finally, the linkage disequilibrium (*r*^2^) between the deletion polymorphism and the thirty-nine surrounding SNPs genotyped in the total study population is displayed in Figure 2. The deletion polymorphism exhibited modest LD with SNP rs7457999 (*r*^2 ^= 0.37) and rs13246630 (*r*^2 ^= 0.20).

## Discussion

The rs7903146 SNP in the *TCF7L2 *gene represents perhaps the most important gene polymorphism implicated in type 2 diabetes, since it is a relatively common variant that confers increased risk for diabetes and this association has been replicated across numerous independent samples [7,9,10,13,17,20,36]. In this study, we report a novel deletion polymorphism on chromosome 7q32 that confers a protective effect for diabetes, with homozygous deletion carriers having a 53% reduced risk of diabetes compared to carriers of the non-deleted region. In addition, we demonstrate an increased risk associated with the minor allele of the *TCF7L2 *SNP that is identified primarily in women, but not men of the Family Heart Study. In this sample, the statistical evidence for association to T2D of the homozygous deletion, although it reduces rather than increases risk, was comparable to that of the *TCF7L2 *SNP. Several nearby SNPs in the 7q32 region also show significant association to T2D, and this may represent linkage disequilibrium among these various polymorphisms. Finally, we report that non-diabetic male homozygous deletion carriers had significantly lower fasting glucose levels, suggesting that the risk for T2D may be mediated by reduced glucose levels.

The region implicated by the deletion and SNP polymorphisms reported here is located between the genes *EXOC4 *(NM_021807) and *LRGUK *(NM_144648). Interestingly, *EXOC4 *is a large gene and its product is part of the exocyst complex 70 (Exo70) that assembles at the plasma membrane of adipocytes in response to insulin and has been reported to play a role in docking and tethering the glucose transporter 4 (*GLUT4*) vesicle to the plasma membrane [37,38]. *GLUT4 *accounts for much of the insulin-stimulated glucose transport in muscle and adipose tissue [38,39]. Inoue et al. (2003) report variability in insulin-stimulated glucose uptake with Exo70 variants, and *EXOC4 *was shown to interact with both the Exo70 wildtype and the amino-terminal fragment of Exo70, which may block the insulin-stimulated assembly of exocyst complex at the plasma membrane. *EXOC4 *has also been shown to be involved the initial docking of insulin vesicles to the cell membrane of pancreatic β cells and is thought to play a role in regulating insulin vesicle exocytosis in response to a glucose stimulus [40]. The potential for this gene to influence diabetes and glucose levels prompted us to evaluate the association to these traits. Though the deletion polymorphism does not seem to overlap with *EXOC4*, transcriptional binding elements could exist downstream of the gene within the region of the deletion that influence transcription. Finally, differences in *EXOC4 *transcription levels could affect glucose stimulated insulin release as well as insulin induced cellular uptake of glucose resulting in the decreased fasting plasma glucose levels found for homozygous deletion carriers in non-diabetics.

Findings in other cohorts also support the presence of a gene(s) influencing diabetes risk in this region. Genome-wide associations for diabetes were recently performed by the Wellcome Trust Case Control Consortium (WTCCC) [41] and the Diabetes Genetics Initiative (DGI) [42], and the results are publicly available. We examined association results for the SNPs in the region spanning the EXOC4 gene, the deletion, and the LRGUK gene, which included 202 SNPs in the WTCCC and 128 SNPs in the DGI studies. From the Wellcome Trust results, we identified 37 SNPs with association p-values less than 0.05, and from the DGI, we identified ten SNPs with association p-values less than 0.05. The best p-value identified in the Wellcome Trust results in this region was 0.0004 at rs6963221 in EXOC4. In the DGI results, the best p-value was 0.015 at rs17167492 in LRGUK. These results from two independent samples lend support for polymorphisms in the region influencing diabetes risk.

One limitation of our study is the self-reported diabetes status. Individuals used in our fasting glucose analysis may have been diagnosed with diabetes after FHS study enrollment or may have failed to report themselves as having been diagnosed for diabetes and, therefore, our analysis of fasting glucose may have included undiagnosed diabetics in the non-diabetic study population. Nevertheless, the glucose levels analyzed were unmedicated measurements.

## Conclusion

In this study, we report a large novel deletion polymorphism that is associated with reduced risk for T2D and several SNPs associated with either increased or decreased risk for T2D on chromosome 7q32 located within a widely replicated BMI linkage region [23-32]. In addition, we demonstrate that non-diabetic males that are homozygous for the deletion polymorphism have lower levels of fasting glucose, which may contribute to protection from T2D. Furthermore, when examined together, the deletion polymorphism offers an effect, albeit protective, comparable to the widely replicated risk associated with the SNP rs7903146 within the transcription factor *TCF7L2*. Since these polymorphisms are in an intergenic region, their relation to nearby gene(s) is speculative and further research will need to be conducted to elucidate the mechanism by which they influence risk for T2D. Ultimately, understanding this mechanism(s) could shed light on the poorly understood relationship between obesity and diabetes and may suggest pathways involved in reducing glucose levels and risk for diabetes.

## Abbreviations

NHLBI: National Heart Lung Blood Institute; FHS: Family Heart Study; BMI: Body mass index; T2D: Type 2 diabetes; PPARG: Peroxisome proliferation activated receptor gamma; KCNJ11: Potassium inwardly-rectifying channel J11; TCF7L2: Transcription factor 7-like 2; SNP: Single nucleotide polymorphism; ACE: angiotension-converting enzyme; EXOC4: Exocyst complex component 4; LRGUK: leucine-rich and gaunylate kinase domain; CHD: Coronary heart disease; IRB: institutional review board; PMP22: Peripheral myelin protein 2; C_t_:cycles-to-threshold; DNA: deoxyribonucleic acid; LD: linkage disequilibrium; RT-PCR: Real time Polymerase chain reaction; GEE: General estimating equation; OR: odds ratio; Exo70: exocyst complex 70; GLUT4: glucose transporter 4; WTCCC: Wellcome Trust Case Control Consortium; DGI: Diabetes Genetics Initiative.

## Competing interests

The authors declare that they have no competing interests.

## Authors' contributions

JML and JBW both assisted in the conception of the study, participated in the design of the study, performed statistical analysis and helped to draft the manuscript. SLW carried out molecular genetic analyses for single nucleotide polymorphisms and the evaluation of the size of the deletion. MWN carried out molecular genetic analyses for SNPs and the deletion assay. JCL coordinated the cleaning and management of SNP and deletion polymorphism data. JET performed molecular genetic analyses of the conserved sequence within the deleted region. MAP and IBB conceived the study and participated in its design and coordination. RHM assisted in the conception of the study, participated in its design and coordination, and helped draft the manuscript. All authors read and approved the final manuscript.

## Pre-publication history

The pre-publication history for this paper can be accessed here:

http://www.biomedcentral.com/1471-2350/9/46/prepub
